# Electromagneto squeezing rotational flow of Carbon (C)-Water (H_2_O) kerosene oil nanofluid past a Riga plate: A numerical study

**DOI:** 10.1371/journal.pone.0180976

**Published:** 2017-08-16

**Authors:** Tasawar Hayat, Mumtaz Khan, Muhammad Ijaz Khan, Ahmed Alsaedi, Muhammad Ayub

**Affiliations:** 1 Department of Mathematics, Quaid-I-Azam University 45320, Islamabad, Pakistan; 2 Nonlinear Analysis and Applied Mathematics (NAAM) Research Group, Department of Mathematics, Faculty of Science, King Abdulaziz University, Jeddah, Saudi Arabia; Cape Peninsula University of Technology, SOUTH AFRICA

## Abstract

This article predicts the electromagneto squeezing rotational flow of carbon-water nanofluid between two stretchable Riga plates. Riga plate is known as electromagnetic actuator which is the combination of permanent magnets and a span wise aligned array of alternating electrodes mounted on a plane surface. Mathematical model is developed for the flow problem with the phenomena of melting heat transfer, viscous dissipation and heat generation/absorption. Water and kerosene oil are utilized as the base fluids whereas single and multi-wall carbon nanotubes as the nanomaterials. Numerical solutions of the dimensionless problems are constructed by using built in shooting method. The correlation expressions for Nusselt number and skin friction coefficient are developed and examined through numerical data. Characteristics of numerous relevant parameters on the dimensionless temperature and velocity are sketched and discussed. Horizontal velocity is found to enhance for higher modified Hartman number.

## 1: Introduction

The development of high energy storage technologies is main topic of the researchers and engineers. It is because of highly demands of heating/cooling in industrial processes. It is an indispensable challenge for the researchers to enhance the heat transfer properties of traditional coolants like water, oil and ethylene glycol which have low thermal conductivity. Due to such motivation first attempt made by Choi [1] in this direction is to pronounce the thermal conductivity of traditional liquids by adding nanosized metallic particles. Nanofluid is the combination of basefluid and nanoparticles having diameter (10–100*nm*). Nanoparticles may be metal nitrides (AiN, SiN), oxide ceramics (Al_2_O_3_, CuO), carbide ceramics (Sic, Tic), metals (Cu, Ag, Au) and carbon (diamond, graphite, carbon nanotubes and fullerene). Hayat el al. [2] discussed unsteady flow of viscous magneto nanofluid by an inclined stretching sheet with thermal radiation, double stratification and viscous dissipation. Rashidi et al. [3] developed the numerical solution of MHD mixed convection flow of nanofluid in a sinusoidal wall channel. Turkyilmazoglu [4] studied the thermal behavior of direct absorption solar collector based on alumnia water nanofluid. MHD forced convection flow of nanofluid over a stretching sheet was examined by Sheikholeslami et al. [5]. Shehzad et al. [6] analyzed three dimensional flow of Jeffrey nanofluid over a stretching sheet by taking the combined effects of internal heat generation and thermal radiation. Hayat et al. [7] discussed stagnation point flow of carbon-water nanofluid over an impermeable stretching cylinder with homogeneous-heterogeneous reactions and Newtonian heating. The steady magnetohydrodynamic (MHD) boundary layer flow of Powell-Eyring nanofluid due stretching cylinder is discussed by Hayat et al. [8]. Zheng et al. [9] analyzed radiation heat transfer of a nanofluid by a stretching sheet with velocity and thermal slip conditions saturated with porous medium. Effect of thermal radiation on magnetohydrodynamics flow of nanofluid between two horizontal rotating plates is studied by Sheikholeslami et al. [10]. Shahmohamadi and Rashidi [11] studied the magnetohydrodynamic (MHD) squeezing flow of nanofluid in a rotating porous channel. Few more studies on this topic can be found through the refs [12–30].

Gallites and Lilausis [31] formulate a Riga plate to create and applied magnetic and electric fields which consequently generates Lorentz force parallel to the wall in order to control the flow of fluid. Riga plate consisted of a span wise aligned array of alternating and permanent magnets mounted a plane surface. It can be used for the radiation of an efficient agent, skin friction and pressure drag of submarines by avoiding the boundary layer separation. In this regard the characteristics of laminar fluid flow due to Riga plate has been investigated in various physical aspects. Pantokratoras and Magyari [32] explored the behavior of fluid flow having low electrical conductivity. They analyzed the opposing and aiding phenomena due to Lorentz force. Fluids having high electrical conductivity *σ* (e.g. semiconductor melts, liquid metals) (*σ* ∼ 106*S*/*m*) can be significantly influenced by applying external magnetic fields of strengths of ∼1 Tesla. This concept is used for the control of classical magneto-hydrodynamic flow. However in weakly conducting fluids (e.g. sea water of *σ* ∼ 10 *S*/*m*) the induction of current by an external magnetic field is not enough. Therefore for higher and efficient flows control (EMHD flow control) an external electric field must be applied. Pantokratoras [33] discussed the behavior of Blasius and Sakiadis flow due to a Riga plate. Mixed convective boundary layer flow of nanofluid induced by Riga plate is examined by Adeel et al. [34].

Phenomenon of melting heat transfer has gained the attention of researchers and scientists due to their high utilization in numerous industrial processes. Further this phenomenon becomes more significant over a stretching sheet which has various industrial applications such as preparation of semi-conductor materials, magma solidification, melting of permafrost and thawing of frozen ground etc. Epstein and Cho [35] discussed the steady flow of viscous fluid over a plate with melting heat transfer. Ching and Li [36] investigated the mixed convection flow and melting heat transfer by a vertical plate in a porous medium. Stagnation point flow of carbon nanotubes with melting heat transfer over a variable thicked surface was discussed by Hayat et al. [37]. Das [38] studied melting and radiation effects in flow of viscous fluid over a stretching sheet. Hayat et al. [39] studied the impact of homogenous-heterogeneous reactions in boundary layer flow of nanofluid saturating porous medium in presence of melting heat transfer. Few studies on heat and mass transfer can be found through the refs [40–43].

From the literature survey it is analyzed that researchers have studied the fluids having low electrical conductivity which provides more skin friction and drag force for fluid flow. Our main objective here is to reduce the skin friction or drag force of such fluids by applying an external electric field. Having such fact in mind we have analyzed the characteristics of squeezed flow of carbon-water nanofluid by a Riga plate with melting heat transfer. Heat transfer is explored with heat generation/absorption and viscous dissipation. Single and multi-wall carbon nanotubes are utilized as nanometrial while water and kerosene oil are used as basefluids. The non-dimensionlized governing equations are solved numerically by built in command ND Solve of Mathematica 9 [44–49]. Impact of various pertinent parameters are discussed through graphs. Characteristics of local skin friction coefficient and local Nusselt number corresponding to various pertinent parameters are studied through numerical data.

## 2: Formulation

**Fig 1:**
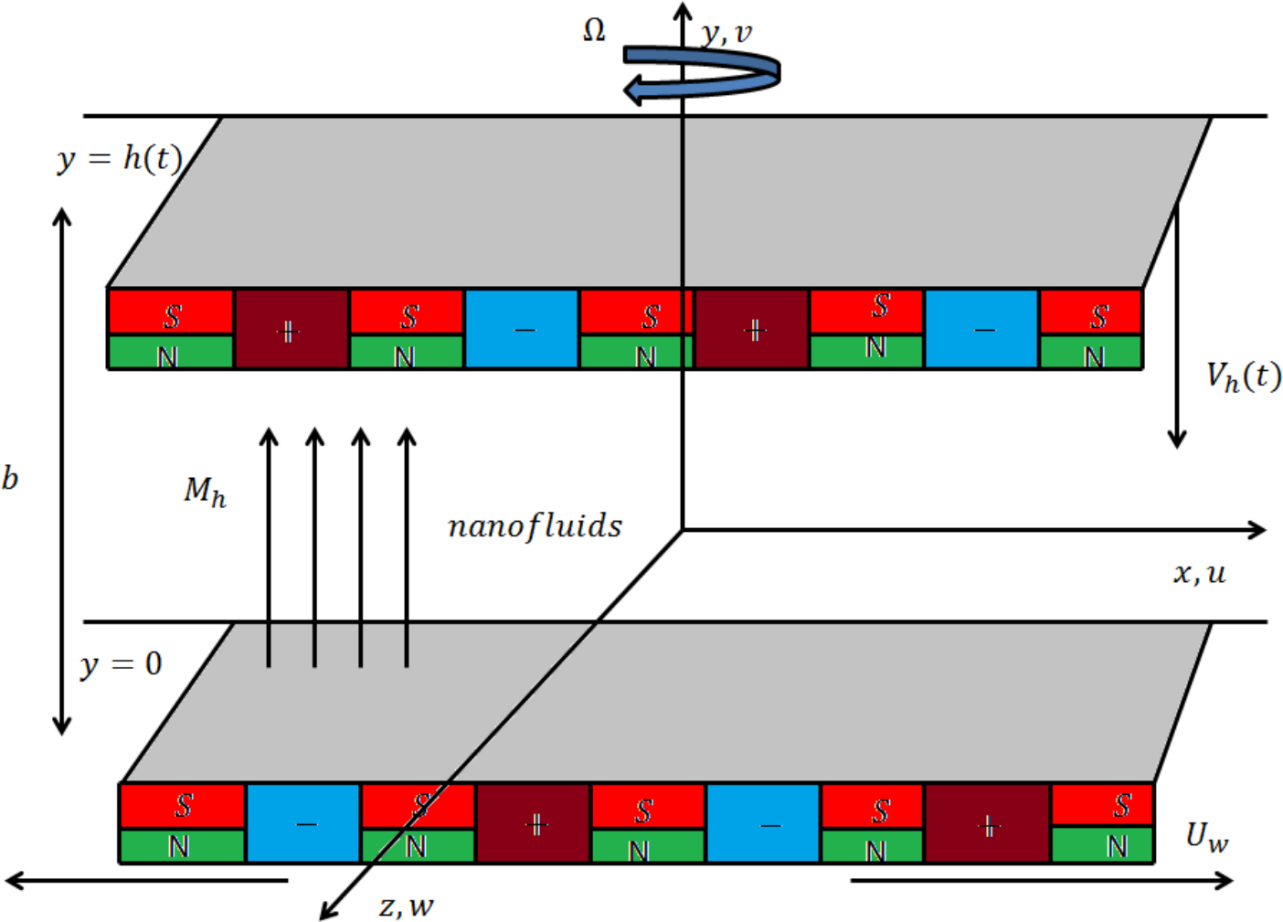
Geometry of flow problem.

We consider the squeezing flow of nanofluid induced by a Riga plate. Single wall carbon nanotube (SWCNT) and multi wall carbon nanotube (MWCNT) are used as the nanomaterials whereas (Water/Kerosene oil) as the base fluid. Cartesian coordinate are selected such that x-axis is in the direction of the stretched Riga plate while y-axis is perpendicular to the x-axis (see Fig 1 Heat transfer characteristics are analyzed by considering viscous dissipation and heat generation/absorption at the surface. More realistic boundary condition in terms of melting heat transfer is imposed. Temperature of the melting surface is assumed less than the ambient temperature. Here fluid with low electrical conductivity is considered. The flow under consideration can be put into the following arrangement [34]:

$$\frac{\partial u}{\partial x}+\frac{\partial v}{\partial y}=0,$$ (1)

$$\left(\frac{\partial u}{\partial t}+u\frac{\partial u}{\partial x}+v\frac{\partial u}{\partial y}+2\frac{\Omega }{1-\gamma t}w\right)=-\frac{1}{\rho_{nf}}\frac{\partial p}{\partial x}+\nu_{nf}\left(\frac{\partial^{2}u}{\partial x^{2}}+\frac{\partial^{2}u}{\partial y^{2}}\right)+\frac{\pi j_{0}M_{0}Exp(-\frac{\pi }{b}y)}{8\rho_{nf}},$$ (2)

$$\left(\frac{\partial v}{\partial t}+u\frac{\partial v}{\partial x}+v\frac{\partial v}{\partial y}\right)=-\frac{1}{\rho_{nf}}\frac{\partial p}{\partial y}+\nu_{nf}\left(\frac{\partial^{2}v}{\partial x^{2}}+\frac{\partial^{2}v}{\partial y^{2}}\right),$$ (3)

$$\left(\frac{\partial w}{\partial t}+u\frac{\partial w}{\partial x}+v\frac{\partial w}{\partial y}-2\frac{\Omega }{1-\gamma t}u\right)=-\frac{1}{\rho_{nf}}\frac{\partial p}{\partial x}+\nu_{nf}\left(\frac{\partial^{2}w}{\partial x^{2}}+\frac{\partial^{2}w}{\partial y^{2}}\right)+\frac{\pi j_{0}M_{0}Exp(-\frac{\pi }{b}y)}{8\rho_{nf}},$$ (4)

$$\left(\frac{\partial T}{\partial t}+u\frac{\partial T}{\partial x}+v\frac{\partial T}{\partial y}\right)=\alpha_{nf}\left(\frac{\partial^{2}T}{\partial x^{2}}+\frac{\partial^{2}T}{\partial y^{2}}\right)+\frac{\mu_{nf}}{{\left(\rho c_{p}\right)}_{nf}}\left(4{\left(\frac{\partial u}{\partial x}\right)}^{{}^{2}}+{\left(\frac{\partial v}{\partial x}+\frac{\partial u}{\partial y}\right)}^{2}\right)+\frac{Q_{0}(T-T_{m})}{{\left(\rho c_{p}\right)}_{nf}},$$ (5)

$$\begin{array}{l} u=U_{w}=\frac{ax}{1-\gamma t},\;v=0,\;w=0,\;T=T_{m}\;at\;y=0,\\ u=0,\;v=v_{h}=\frac{dh}{dt}=-\frac{\gamma }{2}\sqrt{\frac{\upsilon_{f}}{a\left(1-\gamma t\right)}},\;w=0,T=T_{h}\;at\;y=h(t). \end{array}\}$$ (6)

Here *u*, *v* and *w* represent the velocity components in the *x*, *y* and *z* directions respectively, *ρ*_*nf*_ represents nanofluid density, $\nu_{nf}=\left(\frac{\mu_{nf}}{\rho_{nf}}\right)$ nanofluid kinematic viscosity, *μ*_*nf*_ dynamic viscosity, *j*_0_(*A*/*m*^2^) applied current density in the electrodes, *M*_0_(*Tesla*) magnetization of the permanent magnets, *b* width for magnets and electrodes, *Q*_0_ heat absorption /generation coefficients, (*c*_*p*_)_*nf*_ effective heat capacity of nanoparticles, *α*_*nf*_ thermal diffusivity of nanofluid, *T* and *T*_0_ temperature of fluid and solid surface respectively, *a* and *γ* dimensional constants, *k*_*nf*_ the thermal conductivity of nanofluid and Ω for angluar velocity.

Various methods are entailed in the process of solid melting of which these are heat transfers absorption of hidden heat thermophysical properties of a material and the like. The existence of cohesive forces is liable to keep the atoms near to each other in liquid and in solid phases. Molecules move about fixed equilibrium positions in solid materials whereas the same move away in liquid materials. Thus atoms in the liquid materials are more active than those present in the solid materials. It needs a specific amount of energy for solid material to be melted in order to get control of the binding forces that are responsible for solid structure. Such amount of energy is termed as latent/hidden heat of a material. The transfer of a material from one to another phase owing to release or absorption of latent heat takes place at a specific temperature at which the stability of one phase disturbs in favor of another as per the presence of energy [50, 51] i.e

$$k_{nf}{\left(\frac{\partial T}{\partial y}\right)}_{y=0}=\rho_{nf}\left[\lambda_{1}+c_{s}\left(T_{m}-T_{0}\right)\right]v(x,0).$$ (7)

In above relation *λ*_1_ is the latent heat of fluid, *T*_*m*_ and *T*_0_ the melting and solid surface temperatures respectively and *c*_*s*_ represents the heat capacity of solid surface. The melting heat transfer condition i.e. Eq (7) physically represents that heat conducted to the solid surface is the combination of heat of melting and sensible heat required to raise the solid surface temperature *T*_0_ to its melting temperature *T*_*m*_.

Xue [52] analyzed that previous proposed nanofluid models are valid only for spherical or rotational elliptical particles with small axial ratio. Furthermore these models do not describe the properties of space distribution of the CNTs on thermal conductivity. However carbon nanotube can be regarded as rotational elliptical model with large axial ratio. That is why the existing all previous models cannot work on the carbon nanotube based composites. To fill this void Xue [45] proposed a theoretical model based on Maxwell theory including rotational elliptical nanotubes with very large axial ratio and compensating the effects of space distribution on CNTs. The effective properties of carbon nanotubes may be expressed in terms of the properties of the base fluid, carbon nanotubes and solid carbon nanotube volume friction in the base fluid as follows:

$$\begin{array}{l} \mu_{nf}=\frac{\mu_{f}}{{\left(1-\alpha \right)}^{2.5}},\;\rho_{nf}=\left(1-\alpha \right)\;\rho_{f}+\alpha \rho_{CNT},\;\alpha_{nf}=\frac{k_{nf}}{\rho_{nf}{\left(c_{p}\right)}_{nf}},\;v_{nf}=\frac{\mu_{nf}}{\rho_{nf}},\\ \frac{k_{nf}}{k_{f}}=\frac{\left(1-\alpha \right)+2\alpha \frac{k_{CNT}}{k_{CNT}-k_{f}}\ln\frac{k_{CNT}+k_{f}}{2k_{f}}}{\left(1-\alpha \right)+2\alpha \frac{kf}{k_{CNT}-k_{f}}\ln\frac{k_{CNT}+k_{f}}{2k_{f}}},\;{\left(\rho c_{p}\right)}_{nf}=(1-\alpha ){\left(\rho c_{p}\right)}_{f}+\alpha {\left(\rho c_{p}\right)}_{CNT}, \end{array}\}$$ (8)

where *μ*_*nf*_ is viscosity of nanofluid, *α* nanoparticle volume fraction, *ρ*_*f*_ and *ρ*_*CNT*_ the densities of the fluid and carbon nanotubes, *k*_*f*_ and *k*_*nf*_ thermal conductivities of fluid and carbon nanotubes respectively and *ν*_*nf*_ kinematic viscosity of nanofluid.

Transformations are defined as follows:

$$\begin{array}{l} \psi =\sqrt{\frac{a\nu_{f}}{1-\gamma t}}xf(\eta ),\;\eta =\frac{y}{h(t)},\;u=\frac{\partial \psi }{\partial y}=U_{w}f^{\prime }\left(\eta \right),\\ v=-\frac{\partial \psi }{\partial x}=-\sqrt{\frac{a\nu_{f}}{1-\gamma t}}f\left(\eta \right),\;w=U_{w}g(\eta ),\;\theta \left(\eta \right)=\frac{T-T_{m}}{T_{h}-T_{m}}. \end{array}\}$$ (9)

Law of conservation of mass is identically satisfied while Eqs (2–5) take the forms

$$f^{\left(iv\right)}-(1-\alpha +\alpha (\frac{\rho_{{}_{CNT}}}{\rho_{f}})){\left(1-\alpha \right)}^{2.5}(f^{\prime }f^{\prime \prime }-ff^{\prime \prime \prime }+2\omega g^{\prime }+\frac{\beta }{2}(3f^{\prime \prime }+\eta f^{\prime \prime \prime }))-{\left(1-\alpha \right)}^{2.5}M_{h}Ce^{-C\eta }=0,$$ (10)

$$g^{\prime \prime }+(1-\alpha +\alpha (\frac{\rho_{{}_{CNT}}}{\rho_{f}})){\left(1-\alpha \right)}^{2.5}(fg^{\prime }-f^{\prime }g-\beta (g+\frac{\eta }{2}g^{\prime })+2\omega f^{\prime })-{\left(1-\alpha \right)}^{2.5}M_{h}e^{-C\eta }=0,$$ (11)

$$\left(\frac{\frac{k_{nf}}{k_{f}}}{\left(1-\alpha +\alpha \frac{{\left(\rho c_{p}\right)}_{CNT}}{{\left(\rho c_{p}\right)}_{f}}\right)}\right)\;\theta^{\prime \prime }-\frac{1}{2}\beta \eta \mathrm{Pr}\theta^{\prime }+\mathrm{Pr}f\theta^{\prime }+\frac{\mathrm{Pr}Ec}{{\left(1-\alpha \right)}^{2.5}}(4\delta^{2}f^{\prime 2}+f^{\prime \prime 2})+\mathrm{Pr}\phi \theta =0,$$ (12)

with

$$f^{\prime }(0)=1,\;f(1)=\frac{\beta }{2},\;f^{\prime }(1)=0,\;\theta \left(0\right)=0,\;\theta \left(1\right)=1,\;g(0)=0,\;g(1)=0,$$ (13)

$$\mathrm{Pr}\left(1-\alpha +\alpha \frac{\rho_{CNT}}{\rho_{f}}\right)\;f(0)+M\frac{k_{nf}}{k_{f}}\theta^{\prime }(0)=0,$$ (14)

where *α* represents nanoparticles volume fraction, *ω* the rotation parameter, *β* the squeezing parameter, *M*_*h*_ the modified Hartman number, Pr Prandtl number, *Ec* Eckert number, M the melting parameter, *δ* and C the dimensionless parameters, *ϕ* heat generation/absorption and *k* the thermal radiation parameter. These definitions are

$$\begin{array}{l} \;\mathrm{Pr}=\frac{\mu_{f}c_{p}}{k},\;\phi =\frac{Q_{0}(1-\gamma t)}{{\left(\rho c_{p}\right)}_{f}a},\;M=\frac{{\left(c_{p}\right)}_{f}(T_{h}-T_{m})}{\lambda +cs(T_{m}-T_{0})},\\ Ec=\frac{a^{2}x^{2}}{{\left(c_{p}\right)}_{f}(T_{h}-T_{m}){\left(1-\gamma t\right)}^{2}},\;\beta =\frac{\gamma }{a},\;M_{h}=\frac{\pi_{j}M_{0}x}{8\rho_{f}U_{w}^{2}},\\ \;C=\frac{\pi h(t)}{b},\;\delta =\sqrt{(1-\gamma t)\frac{\nu_{f}}{a}}\frac{1}{x}. \end{array}\}$$ (15)

It is noted that *M* is a combination of the Stefan numbers (*c*_*p*_)_*f*_(*T*_*h*_−*T*_*m*_)/*λ*_1_ and (*c*_*s*_)_*f*_(*T*_*m*_−*T*_0_)/*λ*_1_ of liquid and solid phases respectively. If *M* = 0 there is no melting phenomenon and for *α* = 0 the above result is reduced to simple base fluid and there is no nanoparticles.

Skin friction coefficient *C*_*fx*_ and local Nusselt number *Nu*_*x*_ are defined as follows:

$$C_{fx}=\frac{\tau_{w}}{\rho_{f}U_{w}^{2}},\;Nu_{x}=\frac{xq_{w}}{k_{f}(T_{h}-T_{m})},$$ (16)

where the wall shear stress *τ*_*w*_ and the wall heat flux *q*_*w*_ are

$$\tau_{w}=\mu_{nf}{\left(\frac{\partial u}{\partial y}\right)}_{y=0},\;q_{w}=-k_{nf}{\left(\frac{\partial T}{\partial y}\right)}_{y=0}.$$ (17)

In dimensionless form these quantities are expressed as follows:

$$C_{f}Re_{x}^{1/2}=\frac{1}{{\left(1-\alpha \right)}^{2.5}}f^{\prime \prime }(0),\;Nu_{x}Re_{x}^{-1/2}=-\frac{k_{nf}}{k_{f}}\theta^{\prime }\left(0\right),$$ (18)

where Re_*x*_ = *U*_*w*_*x*/*ν* is the local Reynolds number.

## 3: Solution methodology

In this paper we use the numerical built in shooting method. With the help of this method Eqs (10)–(12) are solved with the corresponding boundary conditions given in (13) and (14).

## 4: Discussion

**Fig 2:**
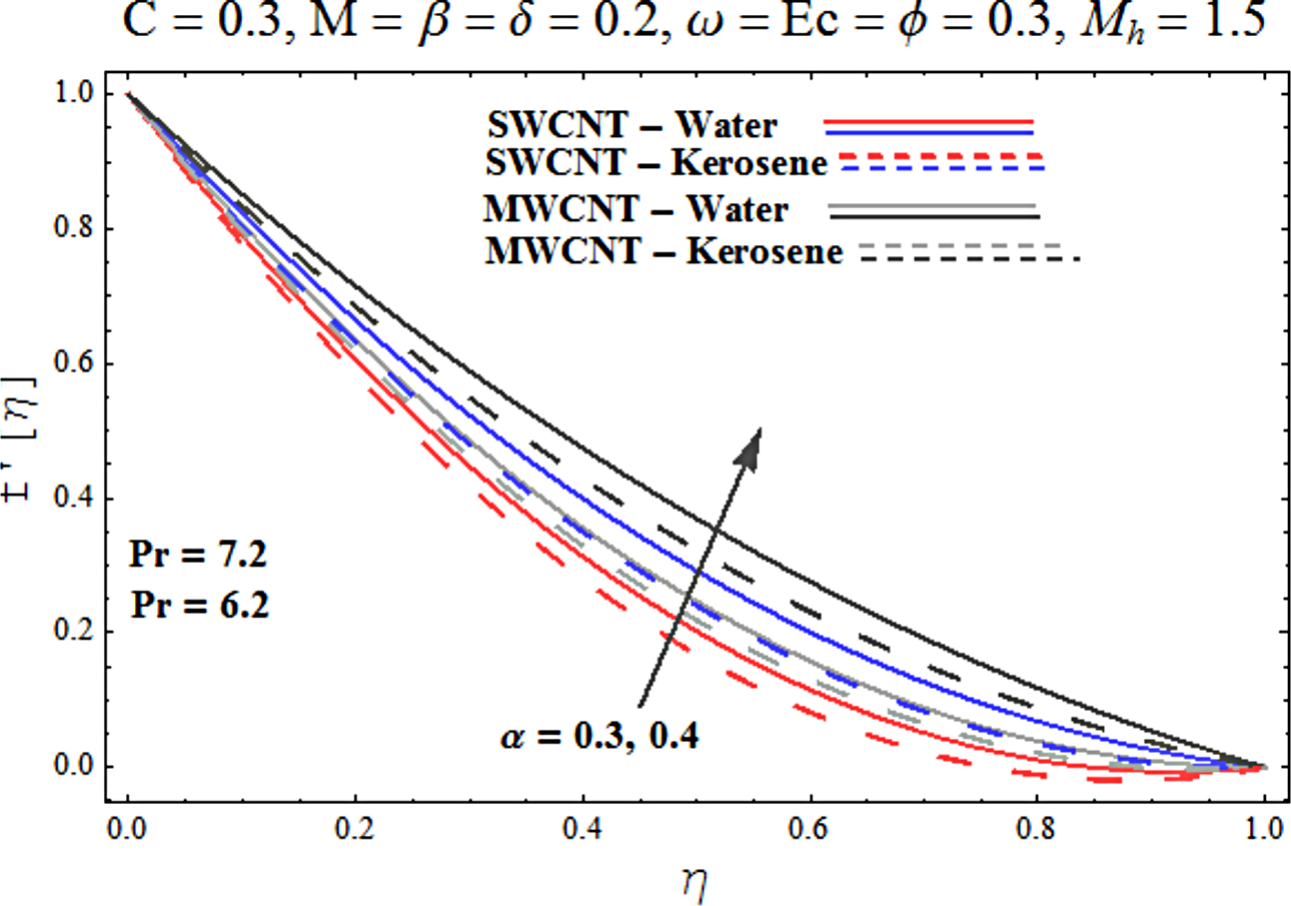
Effect of α on f′.

**Fig 3:**
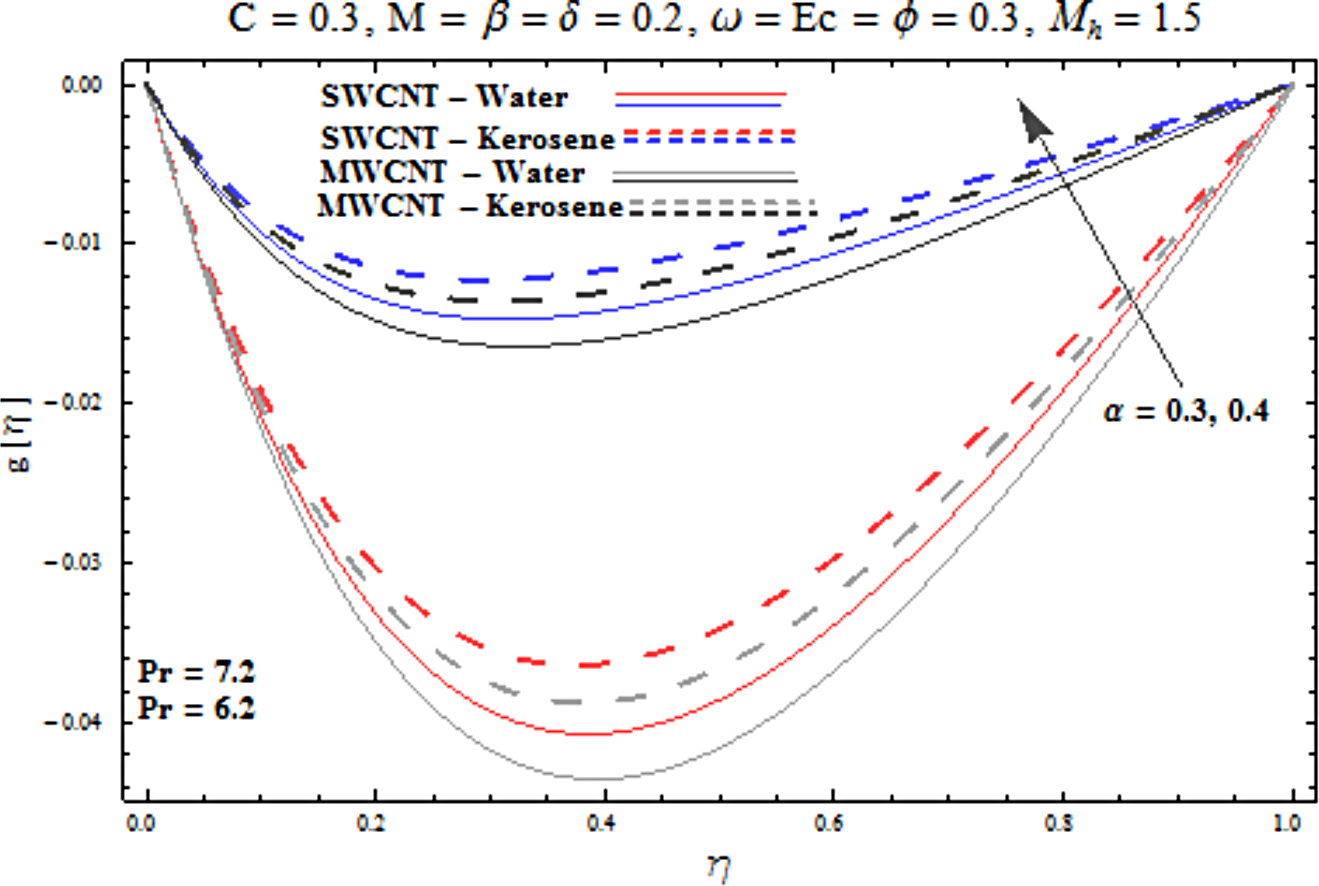
Effect of α on g′.

**Fig 4:**
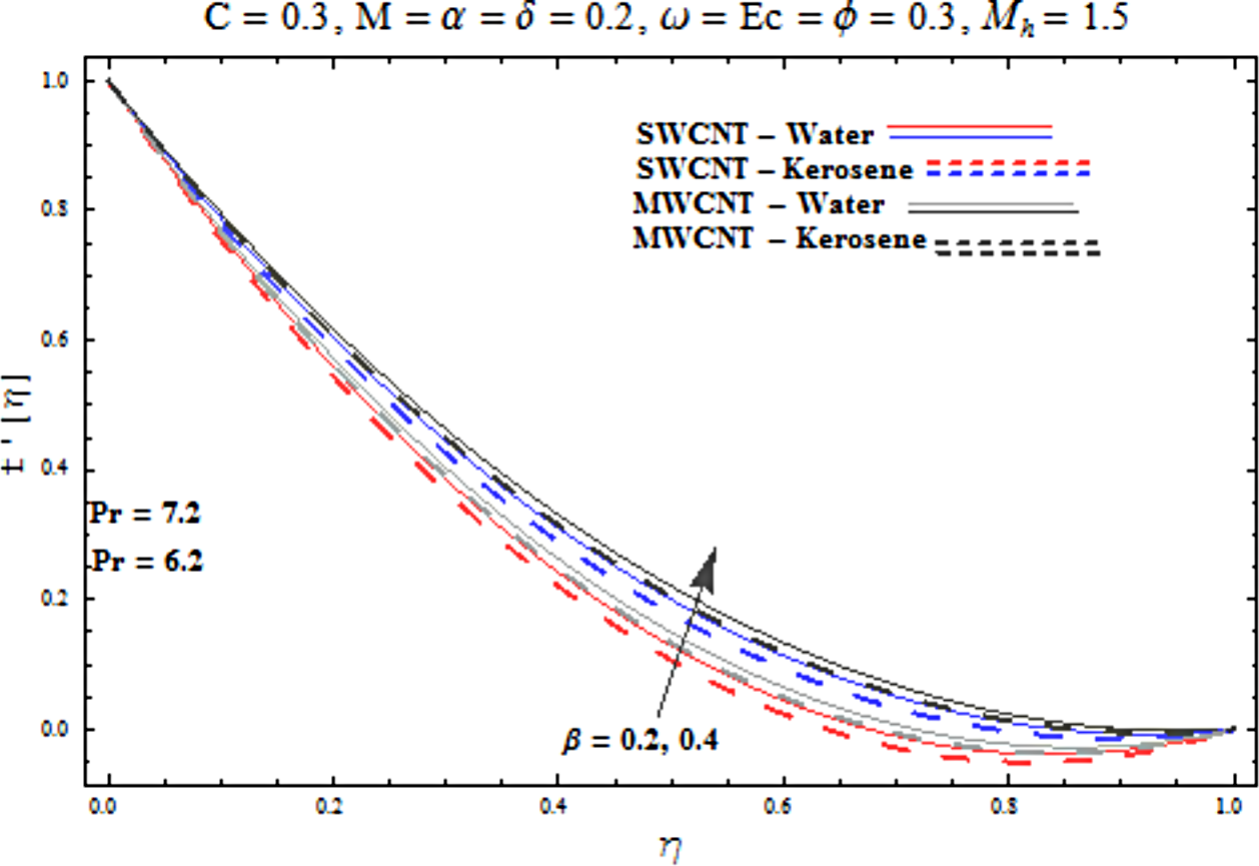
Effect of β on f′.

**Fig 5:**
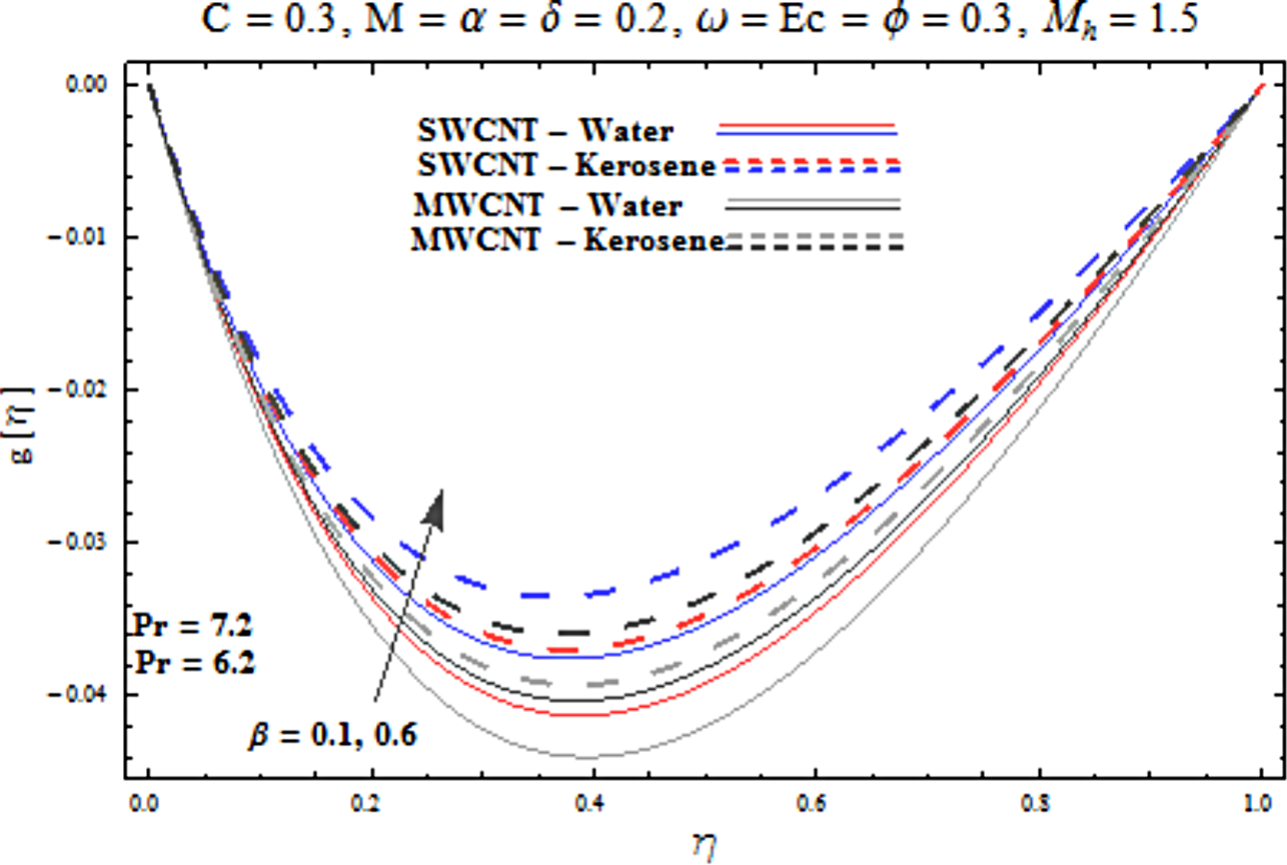
Effect of β on g′.

**Fig 6:**
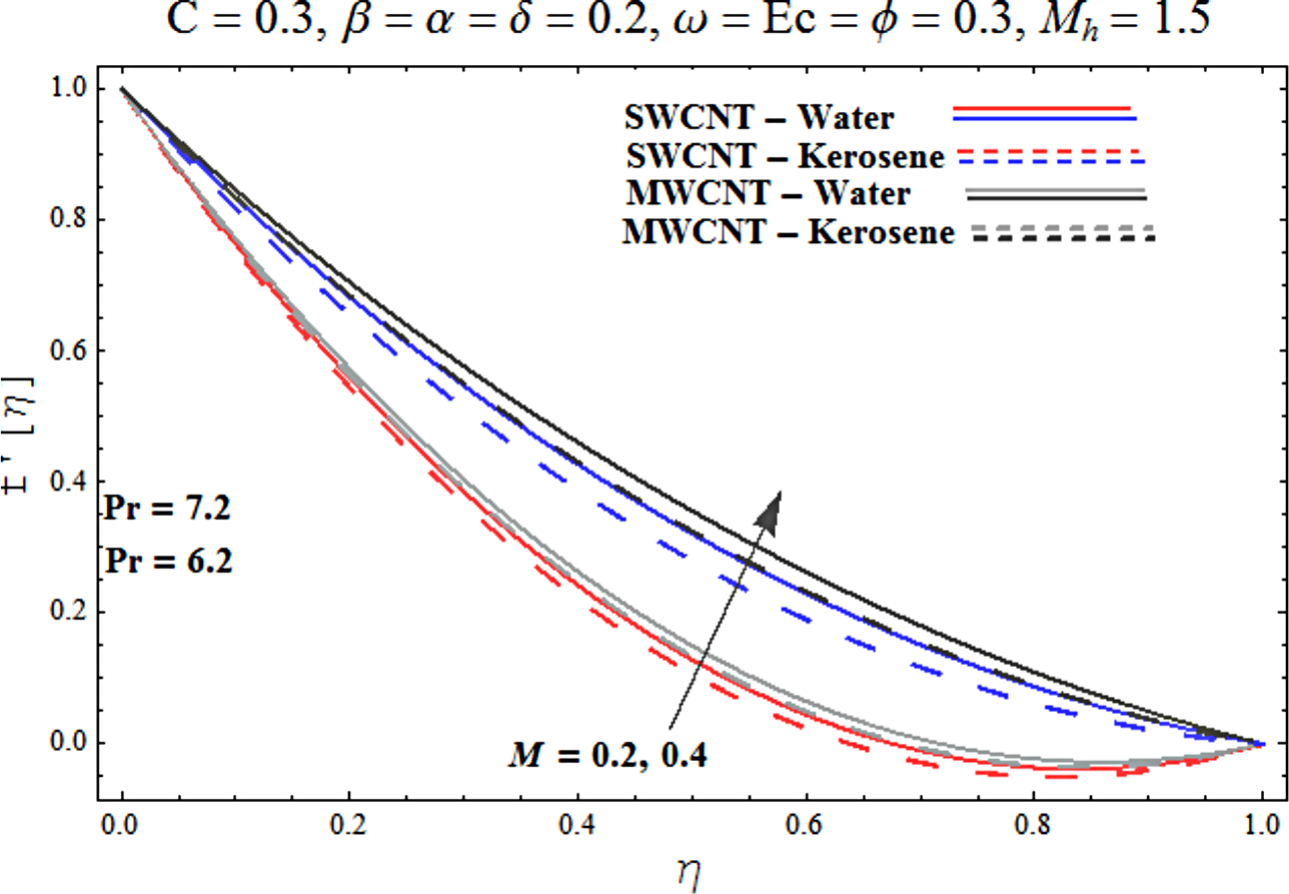
Effect of M on f′.

**Fig 7:**
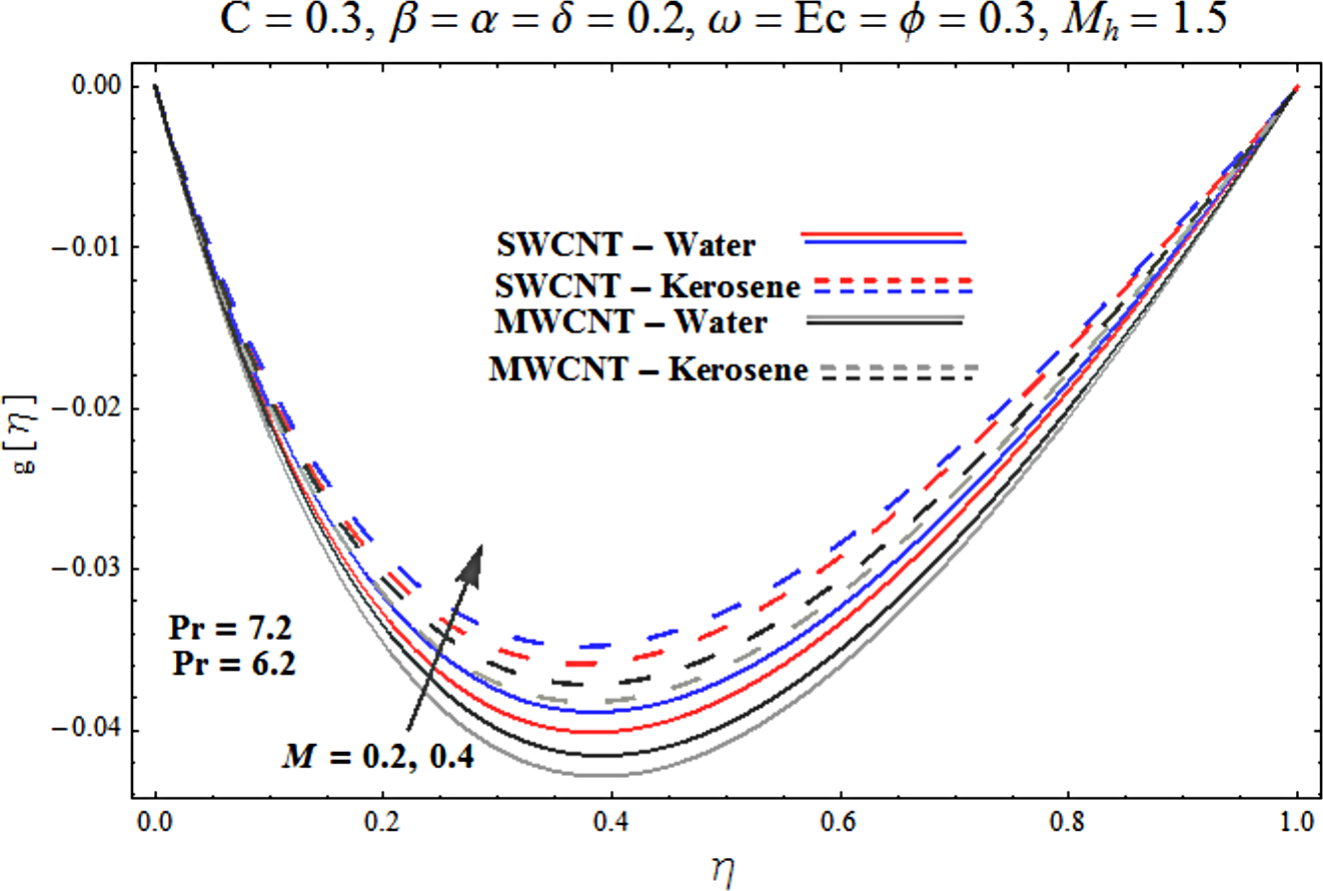
Effect of M on g′.

**Fig 8:**
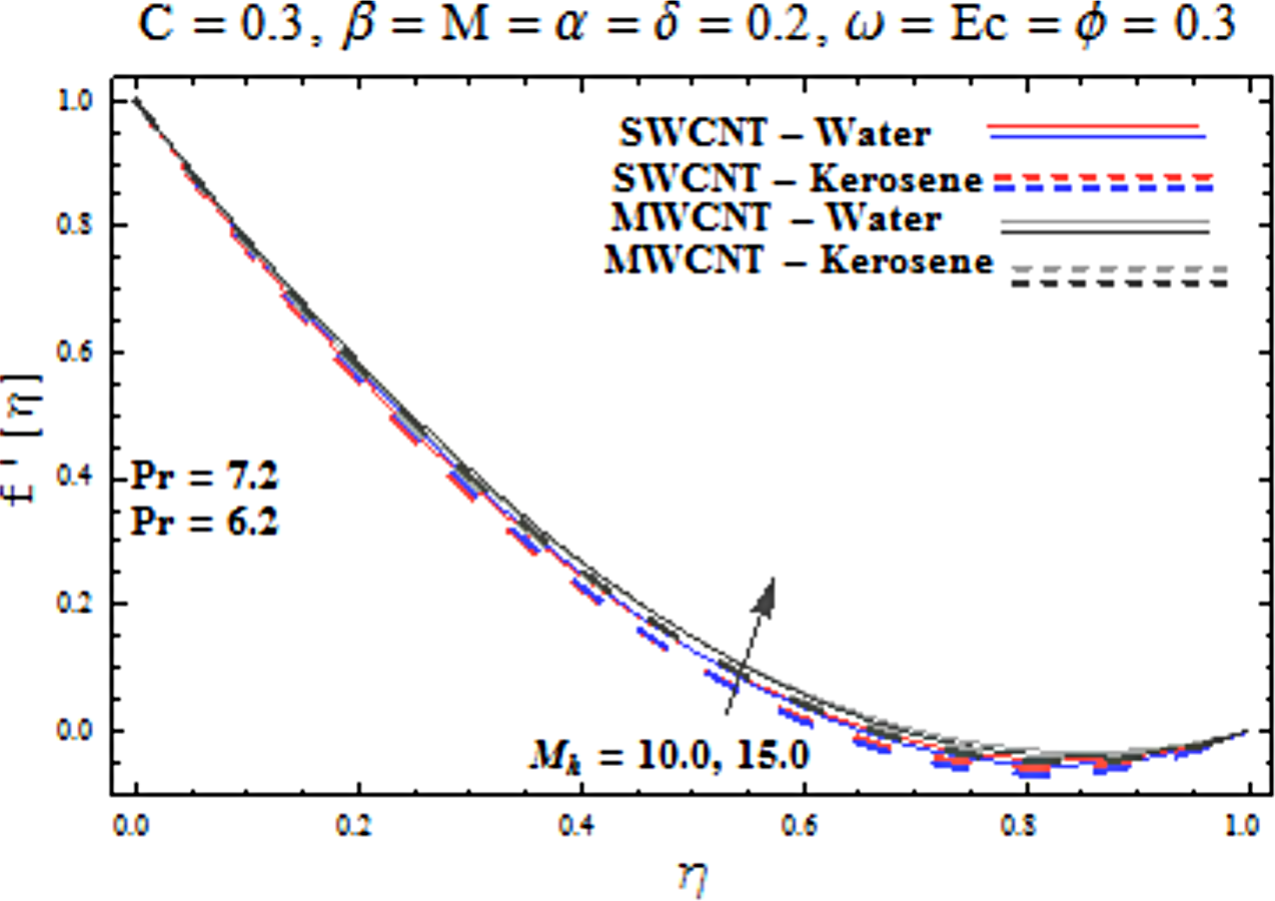
Effect of M_h_ on f′.

**Fig 9:**
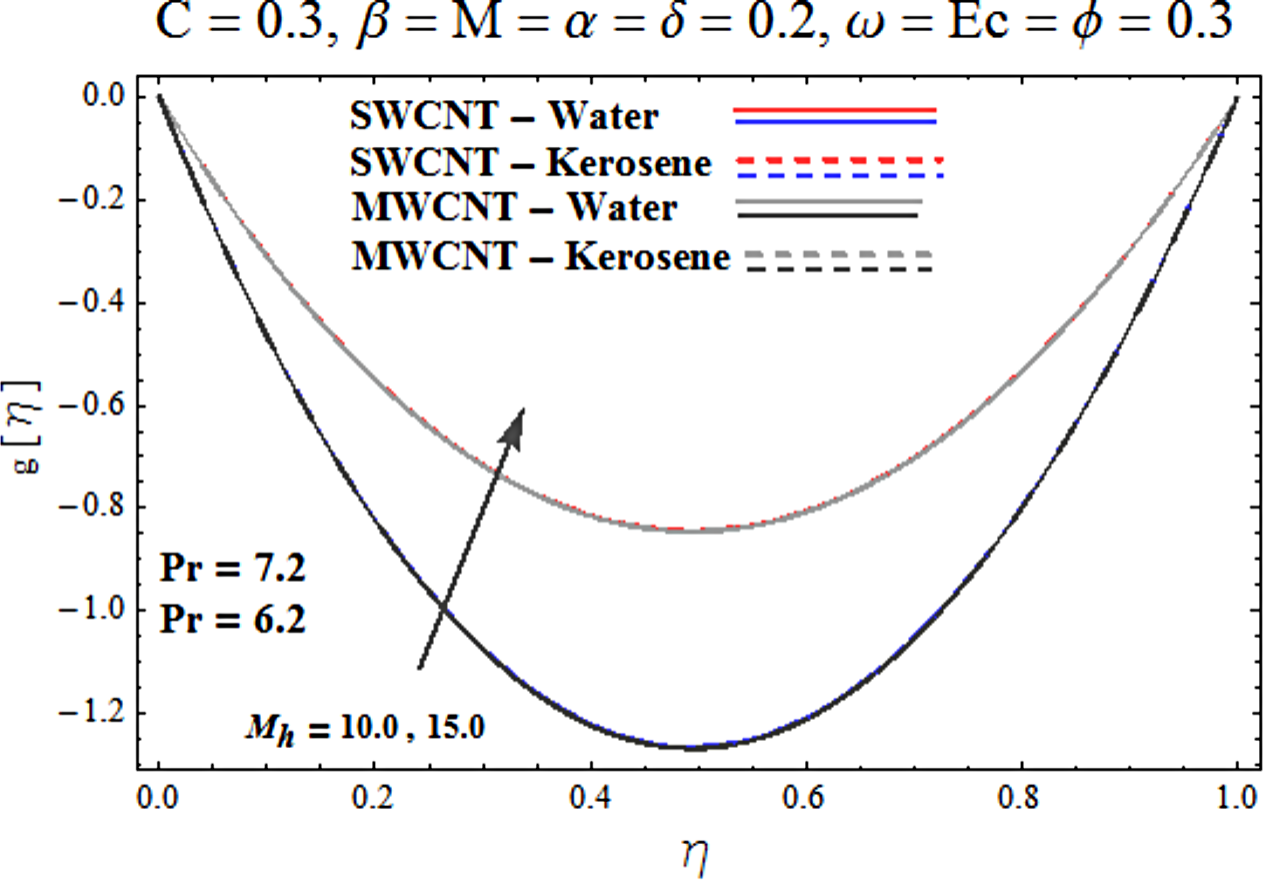
Effect of M_h_ on g′.

**Fig 10:**
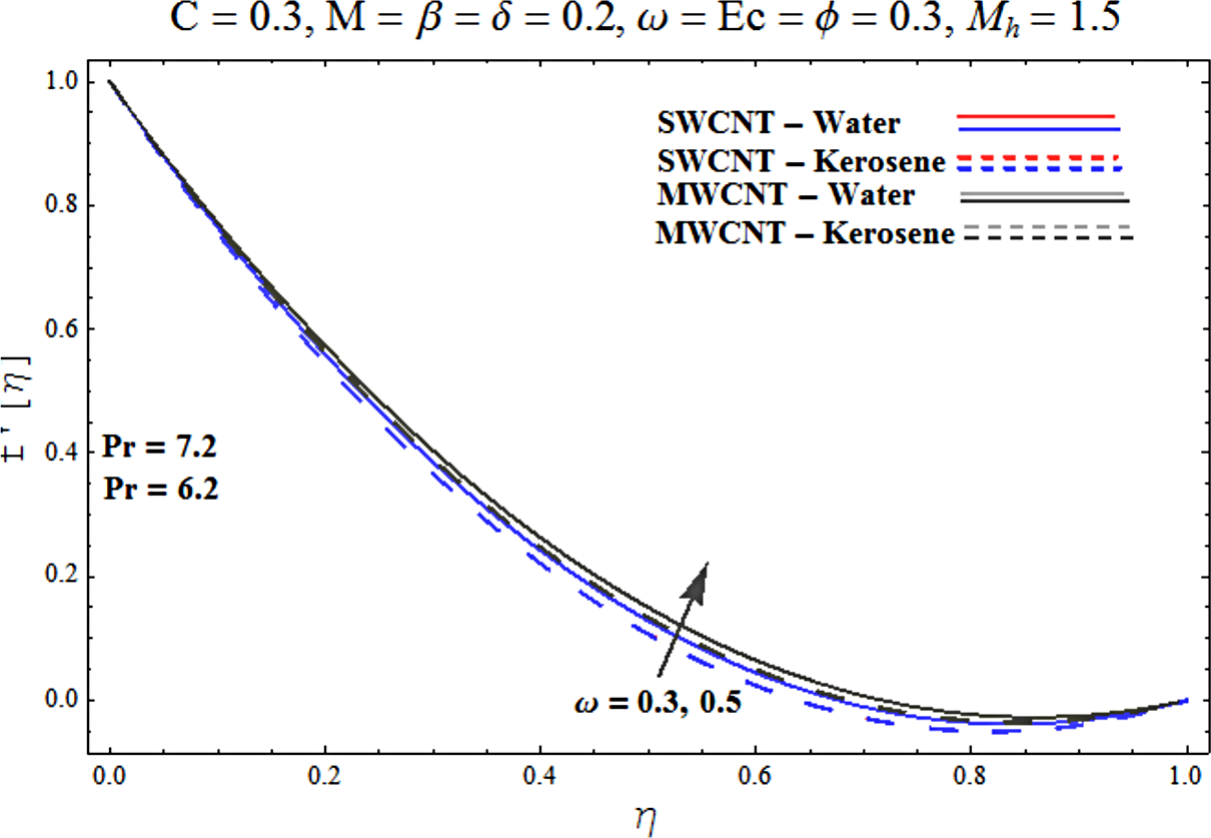
Effect of ω on f′.

**Fig 11:**
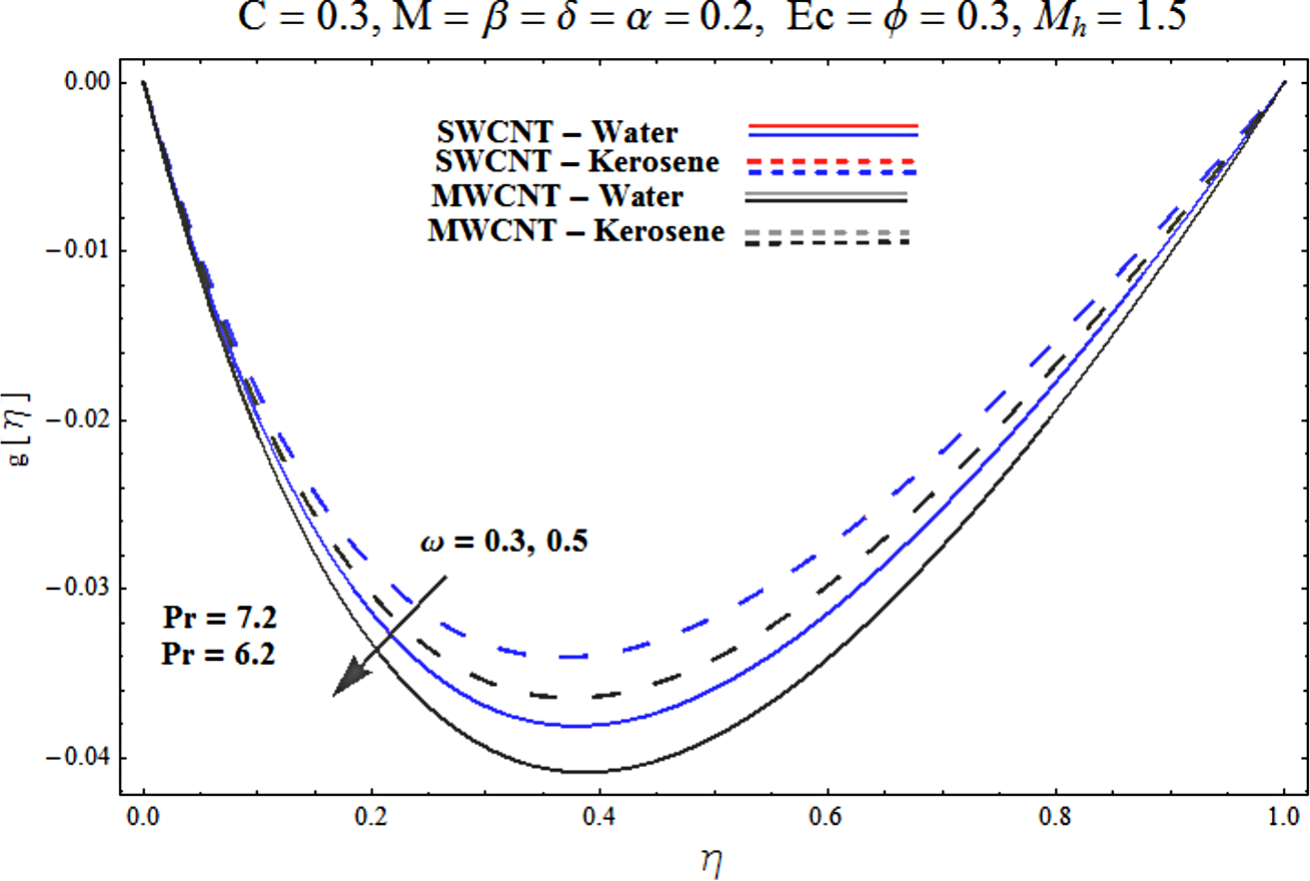
Effect of ω on g′.

**Fig 12:**
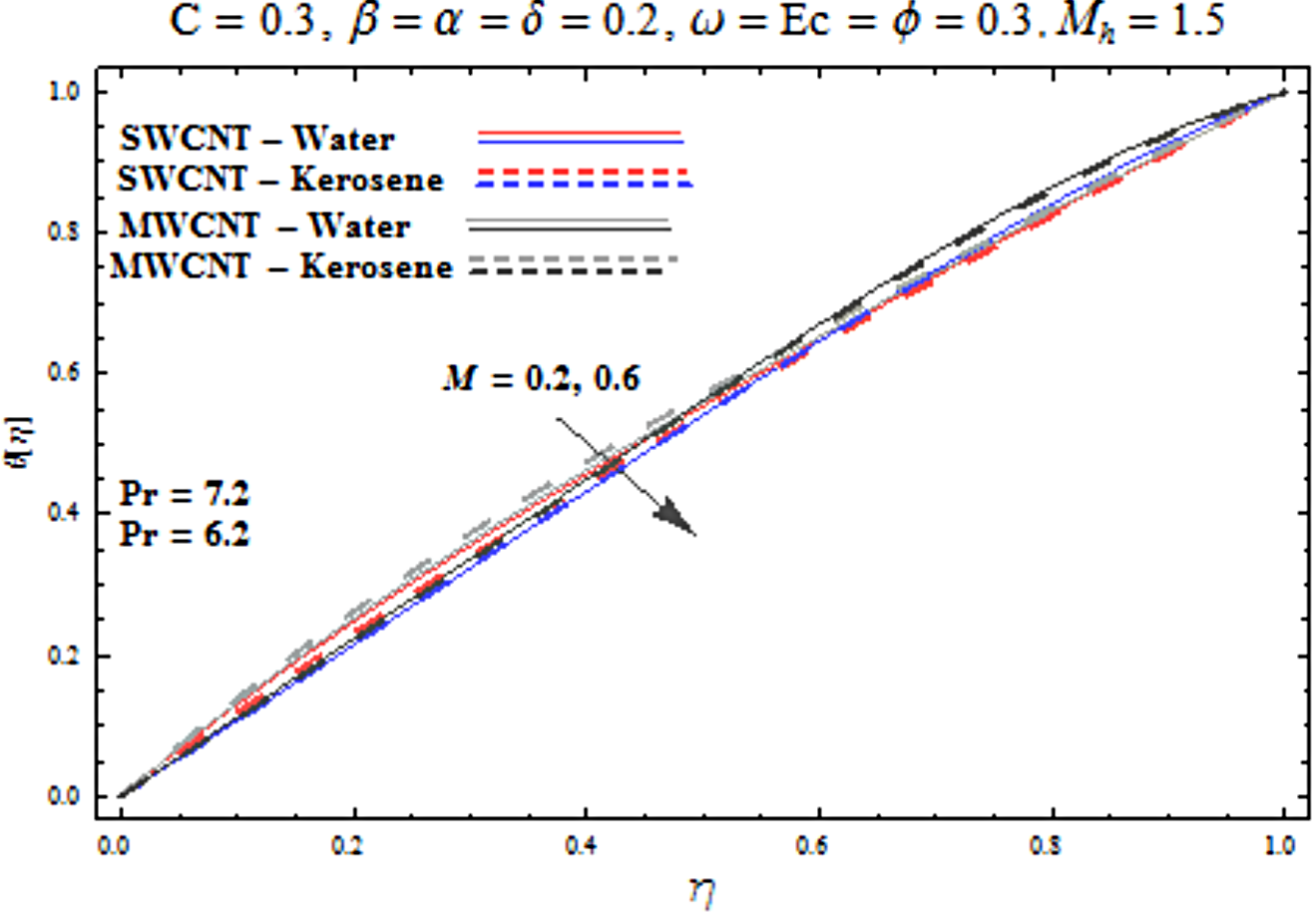
Effect of M on θ.

**Fig 13:**
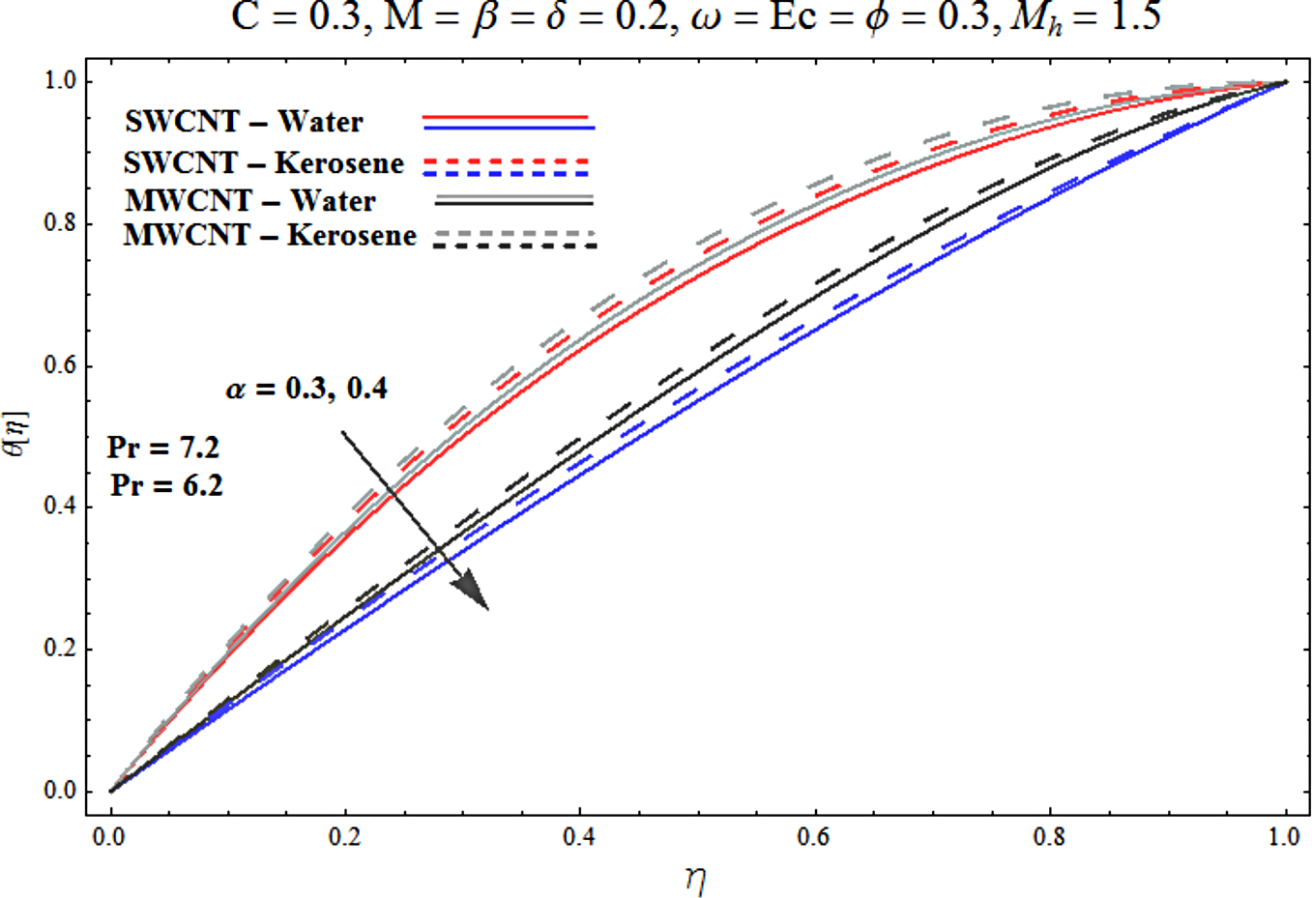
Effect of α on θ.

**Fig 14:**
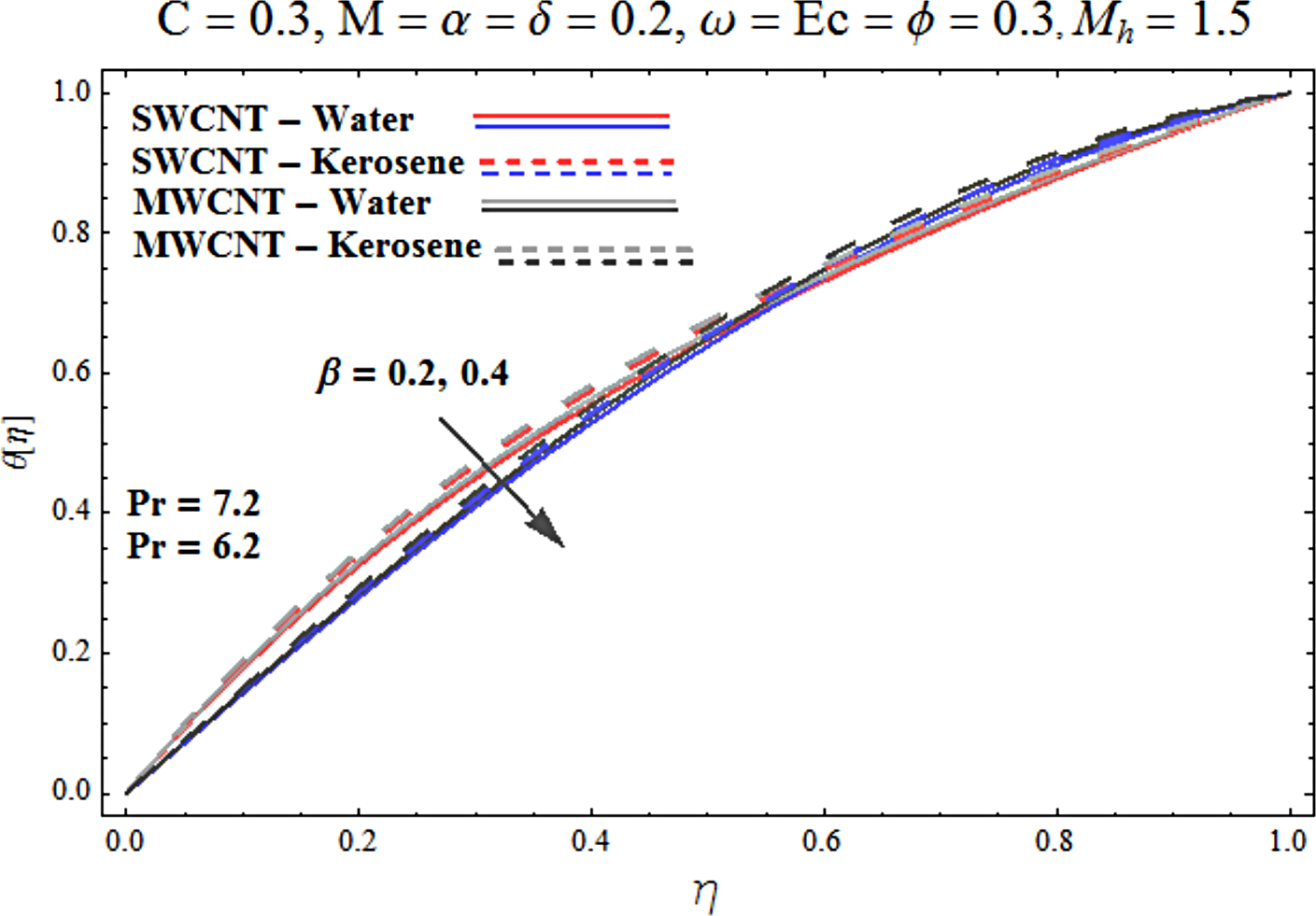
Effect of β on θ.

**Fig 15:**
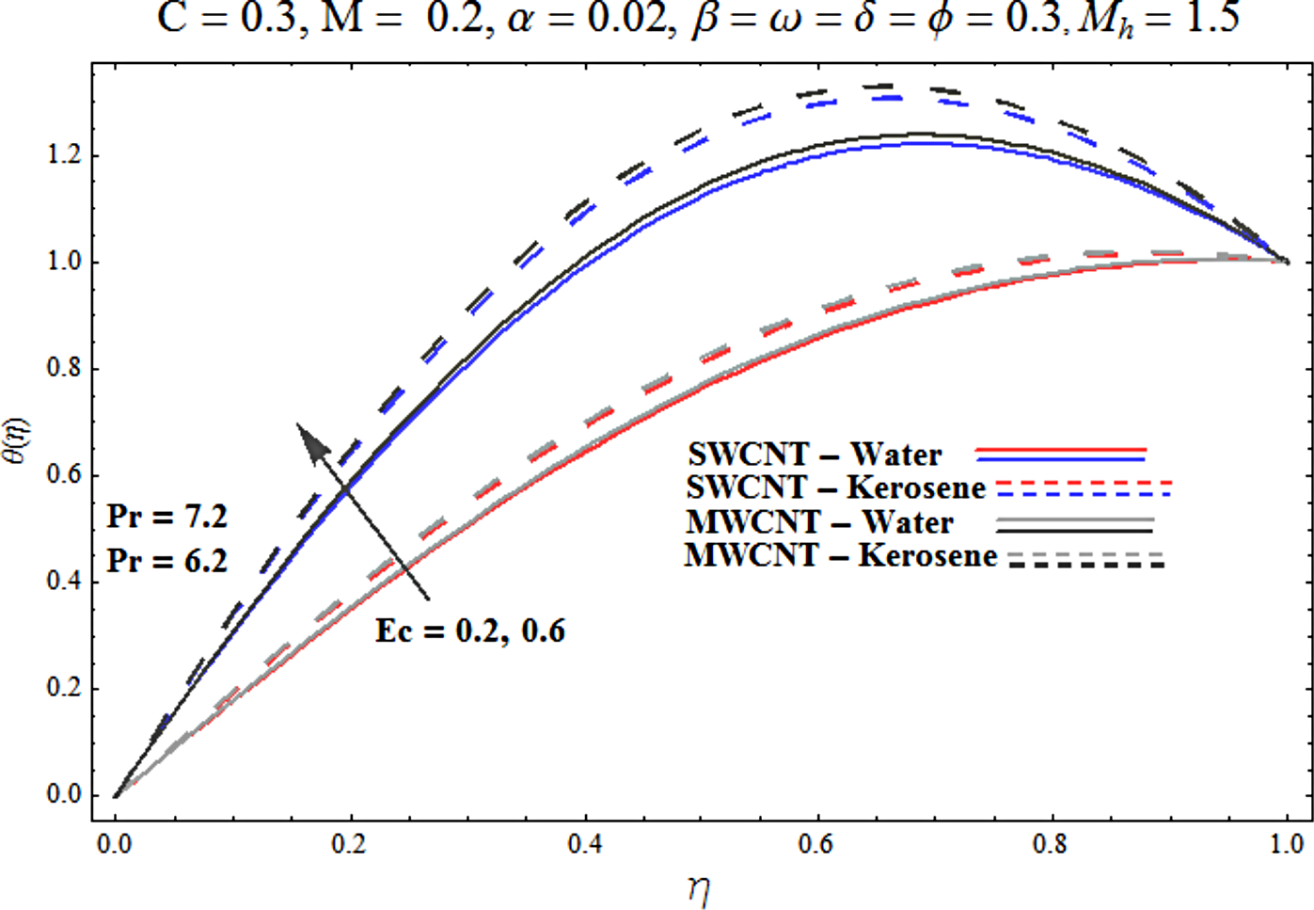
Effect of ϕ on θ.

Main motive here is to describe the features of various physical parameters on the velocity and temperature distributions. Fig 2 reveals the behavior of nanoparticles volume fraction *α* on the velocity profile. It is analyzed that velocity profile shows increasing behavior for higher values of nanoparticles volume fraction for the cases of both single and multiwall carbon nanotubes corresponding to water and kerosene oil base fluids. Further it is noted that velocity distribution dominates in carbon-water nanofluid than carbon-kerosene oil for both SWCNT and MWCNTs. Horizontal velocity is higher at the surface of lower wall when compared to the upper wall. Fig 3 demonstrates the behavior of nanoparticle volume fraction *α* on the transverse velocity distribution. Higher transverse velocity is noted corresponding to small nanoparticle volume fraction for carbon-water and kerosene oil nanofluids. However velocity distribution is prominent for the case of carbon-water when compared with carbon-kerosene nanofluid. Reverse flow is more prominent for smaller values of nanoparticle volume fraction specifically near the lower plate. Characteristics of squeezing parameter *β* on the horizontal velocity distribution is displayed in Fig 4. Velocity distribution augments for higher values of squeezing parameter. Higher velocity of fluid is observed in the vicinity of lower plate due to stretching phenomenon. Further carbon-water has dominating contribution for enhancement of velocity distribution in comparison to carbon-kerosene nanofluid. Behavior of squeezing parameter *β* on the transverse velocity profile is presented in Fig 5. It is analyzed that transverse velocity profile shows increasing behavior with an increment in squeezing parameter. Multi-wall carbon nanotubes have prominent velocity profile than single-wall carbon nanotubes for the case of water and kerosene oil base fluids. Higher reversible flow is noted corresponding to small values of squeezing parameter. Fig 6 represents the impact of melting parameter *M* on the horizontal velocity profile for the case of carbon-water and carbon-kerosene oil nanofluids. Velocity distribution enhances for higher values of melting parameter *M*. In fact an increment in melting parameter corresponds to higher convective flow towards the melting surface which is responsible for enhancement of velocity distribution. Multi-wall carbon nanotubes have dominating behavior in comparison to single-wall carbon nanotubes for both water and kerosene base fluids. However opposite trend is observed for transverse velocity corresponding to higher values of melting parameter (see Fig 7). Variation of modified Hartman number *M*_*h*_ on horizontal and transverse velocity distributions are displayed in the Figs 8 and 9 for both carbon-water and carbon-kerosene oil nanofluids. It is depicted that higher modified Hartman number results in enhancement of horizontal and vertical velocity distributions. Figs 10 and 11 demonstrates the behavior of rotation parameter *ω* on the horizontal velocity distributions *f*′ *and g*′ for single and multi-wall carbon nanotubes. Horizontal velocitiy show increasing behavior for higher values of rotation parameter *ω*. Further higher reverse flow is observed for large rotation parameter. Rotation parameter *ω* has dominating impact on the horizontal distribution near the lower plate. Characteristics of melting parameter *M* on temperature profile is illustrated in Fig 12. It is analyzed that temperature profile decreases with an increment in melting parameter *M* for both single and multi-walls carbon nanotubes. Physically it justifies that as we increase the melting parameter the heat transfers more rapidly to the melting surface due to convective flow. This leads to ultimate decrease in temperature profile. Analysis of nanoparticle volume fraction *α* on temperature distribution is displayed in Fig 13. Temperature profile decreases for larger nanoparticle volume fraction. Temperature profile dominants for multi-wall carbon nanotubes. Fig 14 shows the effect of *β* on the temperature profile. Here Temperature profile reduces. Effect of heat generation/absorption coefficient *ϕ* on the temperature profile *θ*(*η*) is shows in Fig 15. It shows that temperature is an increasing function of *ϕ*. Table 1 is made to show the thermophysical characteristics of base fluid and nanoparticles. Table 2 is arranged to explore the local Nusselt number for various values of pertinent parameters. Here the local Nusselt number is higher for larger nanoparticles volume fraction parameter *α* for both SWCNT and MWCNT cases. Table 3 presents the numerical data of skin friction coefficient for various values of embedded parameters. It has been observed that the skin friction coefficient is higher when the larger values of (*α*) are accounted for both SWCNT and MWCNT.

**Table 1:** Thermophysical characteristics of base fluid and nanoparticles (SWCNT and MWCNT)[37].

Physical Properties	Base Fluid		Nanoparticles	
	Water	Kerosene	SWCNT	MWCNT
*ρ*(*kg*/*m*^3^)	997	783	2600	1600
*c*_*p*_(*J*/*kgK*)	4179	2090	425	796
*k*(*W*/*mK*)	0.613	0.145	6600	3000

**Table 2:** Numerical values of the below mentioned parameters for Nusselt number $Nu_{x}Re_{x}^{-1/2}$.

${Nu}_{x}{Re}_{x}^{-\frac{1}{2}}=-\frac{k_{nf}}{k_{f}}\theta \prime (0)$							Water		Kerosene	
*M*_*h*_	*α*	*ω*	*β*	M	Ec	ϕ	SWCNT	MWCNT	SWCNT	MWCNT
1.5	0.01	0.3	0.2	0.2	0.3	0.4	2.51803	2.50491	2.85513	2.84299
1.6							2.51816	2.50504	2.85532	2.84318
1.7							2.51829	2.50517	2.85551	2.84338
	0.02						2.64541	2.61656	2.99652	2.96848
	0.03						2.78721	2.74117	3.16046	3.11466
		0.4					2.51799	2.50486	2.85507	2.84293
		0.5					2.51795	2.50482	2.85501	2.84287
			0.4				2.43542	2.42234	2.66659	2.65462
			0.5				2.36140	2.34837	2.58780	2.57591
				0.3			2.27295	2.32947	2.64715	2.63279
				0.4			2.13722	2.18389	2.47549	2.45973
					0.3		2.58524	2.67538	3.05862	3.04591
					0.4		2.73037	2.83985	3.25480	3.02146
						0.5	2.92430	2.70145	3.11276	2.89211
						0.6	3.14434	2.92528	3.41334	3.19569

**Table 3:** Numerical values of the below mentioned parameters for skin friction coefficient $C_{f}Re_{x}^{1/2}$.

$C_{f}{Re}_{x}^{\frac{1}{2}}$				Water		Kerosene	
*M*_*h*_	*α*	*ω*	M	SWCNT	MWCNT	SWCNT	MWCNT
1.5	0.01	0.3	0.4	-2.59978	-2.65395	-2.63716	-2.79487
1.6				-2.59344	-2.65143	-2.63084	-2.78877
1.7				-2.58710	-2.64891	-2.62451	-2.78267
	0.02			-2.63108	-2.68202	-2.67644	-2.84197
	0.03			-2.65990	-2.70716	-2.71275	-2.88653
		0.4		-2.60171	-2.65423	-2.63909	-2.79487
		0.5		-2.60363	-2.65451	-2.64101	-2.79673
			0.5	-2.44610	-2.50128	-2.49283	-2.48663
			0.6	-2.30634	-2.36260	-2.36216	-2.35596

## 5: Closing remarks

In this article we have investigated the salient features of melting heat transfer in the squeezing flow of carbon nanotubes over a Riga plate with viscous dissipation. The key points are summarized as follows:

- Velocity distribution for multi-wall carbon nanotubes is higher than the single-wall carbon nanotubes with respect to nanoparticle volume fraction *α* and melting parameter *M*.
- Temperature profile is higher for larger values of viscous dissipation for both SWCNT and MWCNT cases.
- Higher values of melting parameter *M* and nanoparticle volume fraction *α* result in reduction of temperature distribution.
- Velocity profile enhances for higher value of squeezing parameter *β*.

## Abbreviations

*u*,*v*,*w*: Velocity components
*j*_0_: Applied current density
*Ω*: Angular velocity
**M*_0_*: Magnetization permanent magnets
*γ*: Dimensional constant
*P*: Pressure
*ρ_nf_*: Density of nanofluid
*α_nf_*: Thermal diffusivity of nanofluid
*ρ_f_*: Density of fluid
*α_f_*: Thermal diffusivity of fluid
*v_nf_*: Kinematic viscosity of nanofluid
*μ_nf_*: Dynamic viscosity of nanofluid
*v_f_*: Kinematic viscosity of fluid
*μ_f_*: Dynamic viscosity of fluid
**Q*_0_*: Heat absorption/generation
*T*: Temperature
*T_m_*: Temperature of melting surface
*a*: Dimensional constant
*T_h_*: Temperature of squeezing wall
*b*: Width between magnets and electrodes
*(*c*_*p*_)_*nf*_*: Effective heat capacity of nanofluid
**T*_0_*: Temperature of solid surface
*c_s_*: Heat capacity
*k_nf_*: Thermal conductivity
*α*: Nanoparticle volume fraction
**λ*_1_*: Latent heat
*ρ_CNT_*: Density of carbon nanotubes
*Ψ*: Stream function
*β*: Squeezing parameter
*ω*: Rotation parameter
*SWCNT*: Single wall carbon nanotube
*M_h_*: Modified Hartman number
*Pr*: Prandtl number
*MWCNT*: Multi wall carbon nanotube
*ϕ*: Heat absorption/generation parameter
*Ec*: Eckert number
*C_fx_*: Skin friction coefficient
*K*: Thermal radiation
*q_w_*: Wall shear stress
*Nu_x_*: Local Nusselt number
*Re_x_*: Local Reynold number
*τ_w_*: Wall heat flux
**x*,*y*,*z**: Space coordinates
*M*: Melting parameter
*δ*: Dimensionless parameter
*C*: Dimensionless parameter
